# The Natural History of Retinal Sensitivity Loss in Diabetic Macular Ischemia over One Year Evaluated by Microperimetry

**DOI:** 10.3390/jcm13082219

**Published:** 2024-04-11

**Authors:** Wei-Shan Tsai, Sridevi Thottarath, Sarega Gurudas, Jinzhi Zhao, Chui Ming Gemmy Cheung, Taffeta Ching Ning Yamaguchi, Andrea Giani, Elizabeth Pearce, Sobha Sivaprasad

**Affiliations:** 1 Moorfields Clinical Research Facility, NIHR Biomedical Research Centre, Moorfields Eye Hospital NHS Foundation Trust, 162 City Road, London EC1V 2PD, UK; wei-shan.tsai@nhs.net (W.-S.T.); s.thottarath@nhs.net (S.T.); s.gurudas@nhs.net (S.G.); 2 Singapore National Eye Centre, 11 Third Hospital Avenue, Singapore 168751, Singapore; zhao.jinzhi@gmail.com (J.Z.); gemmy.cheung.c.m@singhealth.com.sg (C.M.G.C.); 3 Boehringer Ingelheim, Binger Street 173, 55218 Ingelheim am Rhein, Germany; taffeta.yamaguchi@boehringer-ingelheim.com (T.C.N.Y.); andrea.giani@boehringer-ingelheim.com (A.G.); lizzzpearce@gmail.com (E.P.)

**Keywords:** diabetic macular ischemia, microperimetry, diabetic retinopathy, proliferative diabetic retinopathy, retinal sensitivity

## Abstract

**Background/Objectives**: This one-year prospective observational study, conducted at two centers, aimed to report the natural history of retinal sensitivity (RS) loss in diabetic macular ischemia (DMI). **Methods**: Patients with stable-treated proliferative diabetic retinopathy (PDR) were recruited if there was evidence of DMI on optical coherence tomography angiography, defined as a foveal avascular zone ≥ 0.5 mm^2^ or parafoveal capillary dropout ≥ 1 quadrant. The minimal visual acuity required for performing microperimetry (MP) was ≥54 Early Treatment Diabetic Retinopathy Study letters (Snellen equivalent 20/80). The overall RS (oRS) and pointwise sensitivity (PWS) within the 3 × 3 mm macula were assessed at baseline and twelve months. A value <25 decibels (dB) was defined as impaired RS, and a decrease of 2 and 7 dB was regarded as mild and severe loss, respectively. **Results**: A total of 88 patients (97 eyes) were included. No statistically significant MP changes were detected at one year. However, 10% of the cohort lost oRS ≥ 2 dB, and 73% lost ≥2 dB PWS in ≥5 loci, whereas 1% lost oRS ≥ 7 dB, and 4% lost ≥7 dB PWS in ≥5 loci. The foveola and temporal parafovea were the most vulnerable to severe RS loss. Compared to their counterpart, eyes with baseline oRS ≥ 25 dB had significantly more RS loss in the macula and superior parafovea (55% versus 32% and 53% versus 28%, both *p* = 0.01). **Conclusions**: Rather than oRS loss, ≥2 dB loss in PWS in ≥5 loci is a more feasible outcome measure for clinical trials in DMI.

## 1. Introduction

The prevalence of people with diabetes is increasing exponentially worldwide [1]. The prevalence of visual impairment in people with diabetes also continues to be a global health burden, highlighting the need for novel preventive and therapeutic interventions for diabetic retinopathy (DR) [2]. Among all the complications of DR, diabetic macular ischemia (DMI), defined as an enlarged foveal avascular zone (FAZ) or perifoveal capillary loss, may compromise patients’ vision to below 20/50 [3,4,5,6]. Several clinical trials to stabilize or reverse DMI in stable-treated proliferative diabetic retinopathy (PDR) are ongoing (NCT04919499 and 04424290) [7]. However, a definite visual outcome measure in DMI remains to be determined.

Best-corrected visual acuity (BCVA) is the standard visual function endpoint used in ophthalmic clinical trials. However, BCVA only reflects the foveal cone function [8]. In fact, the influence of DMI may extend outside to the parafovea, and microperimetry (MP) may be more sensitive in depicting these changes. We have demonstrated that overall retinal sensitivity (oRS) in MP is an independent measure of visual function in DMI, as it only moderately correlates with BCVA [9]. Also, MP evaluates global and regional defects on a topographic map, allowing precise correlation between the anatomy and function [8,10]. Therefore, MP is proposed to detect early subtle retinal sensitivity (RS) changes before BCVA loss in DMI [10,11,12].

Among all the commercial MP devices, the macular integrity assessment (MAIA) has been predominantly used in clinical trials, as it has the advantage of a wide dynamic range (0 to 36 decibels [dB]) and the capability to detect scotoma in a shorter timeframe [11,13]. However, a cut-off for defining decreased RS has not been well-established. One observational study on 237 healthy subjects aged 10 to 70 reported a median RS of 32.9 dB (interquartile range 1.8 dB) [14]. Other studies have shown that RS decreases with age (−0.6 dB/decade) and distance from the foveal center [13,15]. From its normal population database, a pointwise sensitivity (PWS) < 25 dB is considered abnormal, irrespective of testing location [15,16]. The same cut-off was used to test 134 eyes with type 2 macular telangiectasia, which showed 0.79 sensitivity and 0.70 specificity to detect ellipsoid zone (EZ) loss [17]. Similarly, the areas with RS < 25 dB also co-located with decreased superficial vessel density (SVD) and deep vessel density (DVD) in DR [18].

Our previous study demonstrated that an oRS of <25 dB is associated with decreased SVD, DVD, and disorganization of retinal inner layers (DRILs) [9]. Longitudinally, Hsu et al., proposed using a percent reduced threshold (PRT), defined as the proportion of RS < 25 dB, in age-related macular degeneration (ARMD), as it differentiated intermediate ARMD from a control group at 1 year (67% versus 30.6%, *p* < 0.001) [19]. On the other hand, the Food and Drug Administration (FDA) and the National Eye Institute Glaucoma Clinical Trial Design and Endpoints Symposium advocate that either a change of ≥5 points or preventing a loss of 7 dB across the total visual field is sufficiently clinically significant to be used as a primary endpoint [11,20]. Combining these criteria, Taylor et al. defined responders as improving by ≥7 dB in ≥5 points within the central area in their latest *RPGR*-associated retinitis pigmentosa trials [11,21]. While using a validated trial endpoint is prudent, susceptibility to RS losses differs between diseases; therefore, researchers should adopt customized approaches.

This study aimed to examine: (1) the natural history of RS loss in DMI over one year, in terms of mean change, proportion with severe loss ≥7 dB, and mild loss ≥2 dB; (2) the area most vulnerable to RS loss; and (3) the utility of baseline oRS of ≥25 dB to predict future RS loss, either globally or regionally.

## 2. Materials and Methods

### 2.1. Study Design

This one-year prospective observational study was conducted at Moorfields Eye Hospital and Singapore National Eye Center from December 2019 to September 2023. The study was approved by the United Kingdom Research Ethics Committee (REC 19/NI/0030) and the Singapore Research Ethics Committee (CIRB Ref No.: 2019/2861). All study participants provided written informed consent.

### 2.2. Inclusion Criteria

Patients with stable-treated PDR, BCVA ≥ 54 Early Treatment Diabetic Retinopathy Study (ETDRS) letters (Snellen equivalent 20/80), and evidence of DMI on optical coherence tomography angiography (OCTA) were recruited. As adopted from ETDRS Report 11, DMI was defined as a FAZ ≥ 0.5 mm^2^ or parafoveal capillary dropout ≥ 1 quadrant [22]. Both eyes were recruited if eligible, and participants were examined at baseline and 1 year. There were no limitations on gender or ethnicity during the selection process.

### 2.3. Exclusion Criteria

We excluded patients with a history of intravitreal injections in the past six months and any other condition that might affect vision, including but not limited to co-existing glaucoma, visually disabling cataracts, other retinal vasculopathy, or macular degeneration. Patients with BCVA < 54 ETDRS letters did not undergo MP at baseline visits, considering the visual acuity (VA) required for a valid MP test. Patients with active PDR or center-involved diabetic macular edema (ciDME) presenting in either visit were treated and excluded. Finally, patients with any fixation losses >30% on MP or missing MP at either visit were excluded from the analysis.

### 2.4. Visual Acuity

A trained optometrist, masked to all other test results, measured the BCVA and low-luminance visual acuity (LLVA) after formal refraction. LLVA was measured after putting a 2.0 log unit neutral-density filter before the testing eye. The maximal letters that a patient could read on the ETDRS chart (Precision Vision, Bloomington, IL, USA) at 4 m were recorded.

### 2.5. Microperimetry

**Figure 1:**
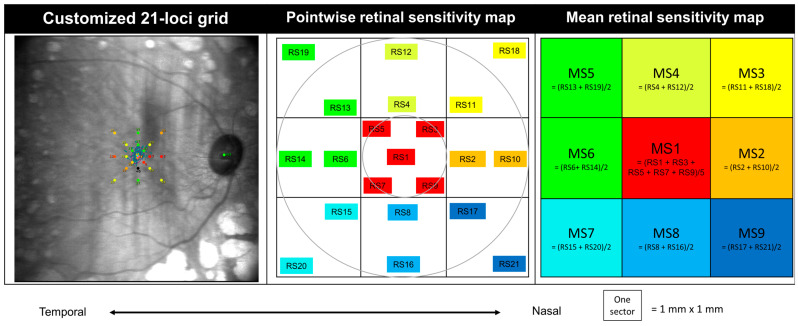
A picture of the 21 testing loci on microperimetry (**left**), the schematic view of these 21 pointwise retinal sensitivities (**middle**) and regional mean retinal sensitivity (**right**). Abbreviations: MS = mean sensitivity; RS = retinal sensitivity.

The RS was assessed on MAIA (CenterVue, Padova, Italy) using a customized grid containing 21 loci within the foveal 3 × 3 mm region (9°) (Figure 1). The oRS was the automatic readout of the mean from the 21 PWS. The parafoveal RS was the mean of RS2, 4, 6, 8, 10, 11, 12, 13, 14, 15, 16, and 17. The mean sensitivity (MS) was the average of the testing loci in that sector with the same color. For example, MS1 was the mean of RS1, 3, 5, 7, and 9.

Calibration of MAIA and testing of reliability were conducted on five normal eyes before recruiting, and the results were within the normative data range according to the manufacturer’s manual. All the participants were pharmacologically dilated and completed a pre-test ten-minute dark adaptation. After that, the patient underwent a training session before the formal testing under mesopic conditions. The right eye, in bilateral cases, or the non-study eye, was examined first.

The settings of the device are summarized below. Goldmann III stimulus size and 200 milliseconds (ms) stimulus duration were selected. The background light intensity was 1.27 candela/m^2^ (4 apostilbs [asb]), and the testing strategy was the standard 4–2 ladder with a range from 0–36 dB. The device was installed with an eye tracker at a speed of 25 Hz (40 ms equivalent), and a follow-up mode was employed at one year.

### 2.6. Outcomes

The primary outcome was the mean change in oRS at one year. For categorical outcomes, a value of <25 dB was defined as decreased RS, regardless of location. Severe and mild RS losses at one year were defined as losses ≥7 dB or ≥2 dB from baseline, respectively. A heat map was generated to depict the most vulnerable loci accordingly. We also assessed the proportion of severe and mild RS losses in ≥5 loci at one year.

### 2.7. Statistical Analysis

Categorical data was summarized using counts and proportions, while continuous data was described using means and standard deviations. A paired *t*-test was used to analyze the changes in the same eye at different time points while correcting for the clustering subject. Given the involvement of both eyes, *p* values for comparing the categorical data were computed using the generalized estimated equation (GEE) method from the binomial family with a logit link. Significance was defined as *p* < 0.05. All statistical analyses were conducted using Stata MP 15.

## 3. Results

A total of 88 DMI patients (122 eyes) completed the baseline and one-year visits. Two eyes developed ciDME, twelve had fixation errors >30%, and eleven could not cooperate with the examinations; therefore, 97 (80%) eligible eyes were included in the final analysis.

### 3.1. Demographics and Ocular Characteristics

The average age of this cohort was 58 ± 12 years old, and the mean duration of diabetes was 27 ± 14 years (Table 1). The mean BCVA was 78 ± 8 ETDRS letters, and 88% had a BCVA of 70 ETDRS letters or more.

### 3.2. Microperimetry Changes in DMI over One Year

**Figure 2:**
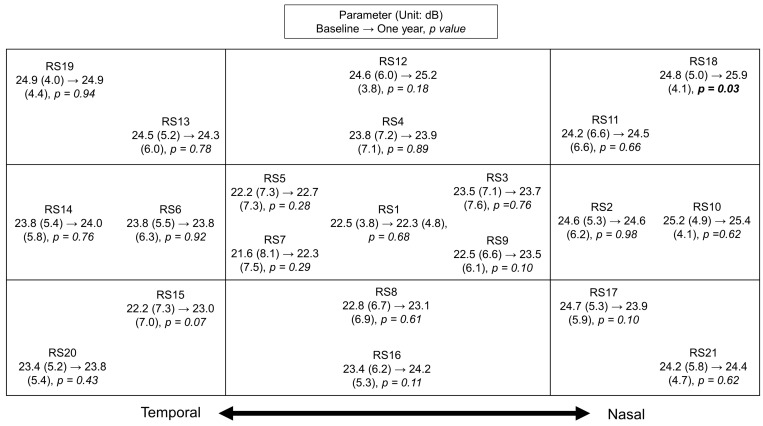
A topographic map showing the retinal sensitivity change from baseline to one year at different loci. There was no significant deterioration in overall, parafoveal, or pointwise retinal sensitivity. Abbreviations: dB = decibels, RS = retinal sensitivity.

Fifty eyes (52%) in the cohort had a mean oRS of <25 dB (Table 2 and Figure 2). There were no significant MP changes in the oRS from baseline to one year (23.7 ± 4.2 to 23.9 ± 3.9 dB, *p* = 0.33). Also, no statistically significant RS deterioration was observed in MS or PWS.

### 3.3. The Severity of RS Losses in DMI

When focusing on absolute RS losses, 43% of the study eyes presented with oRS loss of any degree after one year of observation (Table 3). Among these, 10% had mild oRS loss (≥2 dB); however, only 1% had severe oRS loss (≥7 dB). When considering PWS, the proportion of ≥2 dB varied from 27% to 46%. In contrast, only 1% to 8% of the same cohort showed severe PWS loss ≥7 dB.

### 3.4. The Distribution of RS Losses in DMI

**Figure 3:**
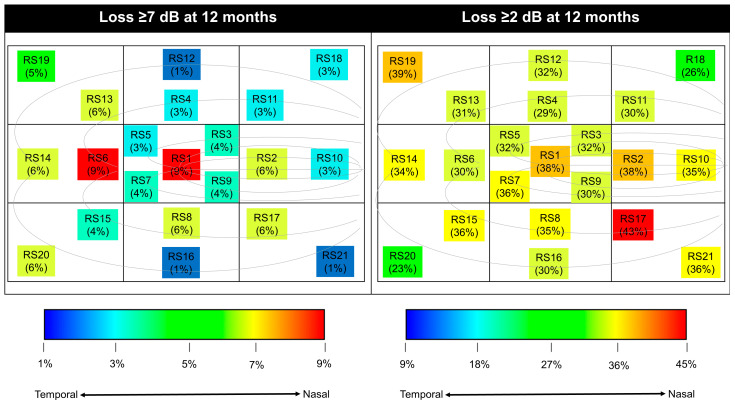
A heat map of the most vulnerable loci at risk of severe and mild retinal sensitivity loss at one year. The curvilinear lines depict the location of papillomacular bundle. Abbreviations: dB = decibel; RS = retinal sensitivity.

On heat map examination (Figure 3), severe PWS loss ≥7 dB at one year was most likely to be found in the center and proximal temporal parafovea (RS1 and RS6). Using the same map to look at mild RS loss ≥2 dB over one year showed that the inferonasal parafovea appeared to be the most susceptible regions (highest at RS17), followed by the foveola.

Next, we evaluated RS losses in ≥5 loci. Only 4% (4 out of 97 eyes) had PWS loss ≥7 dB in ≥5 loci in the macula at one year (Table 3). However, 73% experienced PWS loss ≥2 dB in ≥5 loci. In the parafoveal region where 12 loci were tested, 45% of the eyes exhibited PWS loss ≥2 dB in ≥5 loci.

### 3.5. The Utility of Baseline oRS 25 dB in Predicting Future Development in DMI

We further examined the utility of baseline oRS of 25 dB to detect future RS loss at one year (Table 4). We found that eyes with oRS ≥ 25 dB at initial presentation were almost twice as likely to have oRS loss of any degree at one year compared to their counterparts (55% versus 32%, *p* = 0.01). Eyes with baseline oRS ≥ 25 dB also lost RS more frequently in the superior parafovea (MS4) than those with oRS < 25 dB (53% versus 28%, *p* = 0.01).

In addition, Table 5 shows that eyes with baseline oRS ≥ 25 dB tended to experience PWS loss ≥2 dB in ≥5 loci at one year in the parafoveal region compared to those with oRS < 25 dB (55% versus 36%, *p* = 0.05). The sample size of RS loss ≥7 dB in ≥5 loci was too small to do the same analysis.

## 4. Discussion

This one-year prospective observational study provides several findings for future trial designs on DMI. First, we show that RS losses are mild in DMI with no statistically significant differences in oRS, MS, and PWS at one year compared to the baseline. Hence, detecting subtle RS losses is necessary to evaluate the effects of therapeutic agents within the typical time scale of clinical trials. This concept is highlighted in the second finding from our study: at one year, 10% of the DMI eyes experienced oRS loss ≥2 dB, and 73% had PWS loss ≥2 dB in ≥5 loci in the macula. Severe loss of ≥7 dB is uncommon. A further finding is that the foveola and the temporal parafovea are most vulnerable to severe RS loss of ≥7 dB. Finally, eyes with baseline oRS ≥ 25 dB, compared to their counterparts, had RS loss of any degree more frequently in the macula and superior parafovea at one year (both *p* = 0.01). This finding indicates that RS losses are more likely seen in eyes with presenting oRS ≥ 25 dB. However, the impact of such losses regarding visual function at an individual level is unclear. For example, one could argue that a drop of 2 dB in an eye with a mean oRS of 27 dB may not be functionally impactful.

Compared to normal subjects, most eyes with DMI have significantly reduced oRS [23]. However, a cut-off value to define decreased oRS is lacking. Pereira et al. examined five DMI eyes on MAIA and reported that the oRS across the 10° diameter macula ranged from 10.7 to 12.8 dB [24]. Our previous report demonstrated that attaining or maintaining 25 dB oRS is worth considering as a trial endpoint as it reflects the changes in structural abnormalities without complex calculations and age adjustments [9]. In this paper, we assessed the utility of a baseline oRS of 25 dB to predict future RS loss. We found that eyes with oRS ≥ 25 dB at initial presentation were twice as likely to have oRS loss of any degree at one year compared to their counterparts (55% versus 32%, *p* = 0.01). We also showed eyes with baseline oRS ≥ 25 dB tended to experience RS loss ≥2 dB in ≥5 loci in the parafovea at one year compared to those with oRS < 25 dB (55% vs. 36%, *p* = 0.05). Together, it is better to evaluate RS loss in DMI eyes with oRS ≥ 25 dB. This unique cut-off has been integrated by Hsu et al., in the form of percent reduced threshold (PRT), defined as the percentage of PWS < 25 dB, to predict RS loss in ARMD, and they advocated for PRT as the most robust indicator of ARMD progression [19].

The evolution of RS losses varies in different retinal conditions; therefore, it is necessary to adopt customized criteria for monitoring disease progression [11]. For example, the reduction rate was 0.34 dB/year in *USH2A* retinopathy, and a slightly faster progression was found in Stargardt’s disease (ranging from 0.68 to 1.19 dB/year) [25,26,27]. While an average 0.85 dB/year decline was noted in *CRB1*-associated retinal dystrophies, half of the *RPGR*-associated retinitis pigmentosa exhibited 1.3 dB loss at one year [21,28]. The rate of oRS loss in ARMD is even more aggressive, worsening by 1.4 dB/year and 3.0 dB/year in early and intermediate ARMD [19]. In comparison, the mean change in oRS in DMI was 0.3 dB/year, highlighting the slow progression of the disease. Our cohort also presented with a mild increase in BCVA from 77.9 ± 8.0 letters at baseline to 78.9 ± 8.1 letters at 12 months (*p* = 0.04). Interestingly, those eyes with severe RS loss of ≥7 dB did not have the same level of BCVA loss, echoing our previous report that RS does not correlate well with BCVA (ρ = 0.44, *p* < 0.001) [9].

Taylor et al. showed, in their *RPGR*-associated retinitis pigmentosa study, that 54% of the eyes lost ≥7 dB in ≥5 loci at one year [21]. These eyes bore the potential to meet the successful treatment criteria set by the FDA, i.e., improvement by ≥7 dB in ≥5 loci. However, at twelve months, only 4% of our DMI cohort lost ≥7 dB in ≥5 loci. The result implies severe RS losses in DMI are uncommon over one year.

In the setting of DMI, oRS loss of ≥2 dB/year could be considered a trial endpoint. Our findings suggest that it would be more reasonable to consider ≥2 dB/year oRS loss as a trial endpoint rather than ≥7 dB/year, as 10% of our cohort fulfilled this criterion. Moreover, 73% had RS losses ≥2 dB in oRS in ≥5 random loci by one year, indicating that reducing the proportion of RS losses ≥2 dB in oRS in ≥5 random loci may also be a feasible option in the clinical trials investigating treatment options targeting DMI. The open question, thus, is whether losing ≥2 dB per year is clinically meaningful for DMI. Several DME studies have reported significant improvement of oRS ≥ 2 dB after subthreshold micropulse laser and anti-vascular endothelial growth factor [24,29,30,31,32]. The most significant gain was reported by Pereira et al., in which the oRS of five DMI eyes improved by 5 dB at 24 weeks (*p* = 0.007) after six monthly bevacizumab injections [24]. However, the small sample size could have severely affected the validity of the results.

Another important finding from this study is that the loci most susceptible to ischemic injury in DMI seem to distribute along the inferior papillomacular bundle, as shown in Figure 3. We found that the foveola and the proximal temporal parafovea were the two most frequent points to have severe RS loss of ≥7 dB, whereas the proximal inferonasal parafovea was the number one location with mild RS ≥ 2 dB at one year. This finding corroborates a study by Sim et al., in which they reported that the prevalence of ischemia was 87% and 35% in the temporal and papillomacular areas, respectively, in severe DMI [3]. The temporal parafovea is the watershed zone between the superior and inferior capillary plexus on the horizontal raphe, making it more prone to ischemia [33,34]. Moreover, it has been proposed that the papillomacular bundle axons are slender in caliber compared to other retinal ganglion cell fibers, predisposing them to energy depletion damage [35]. However, the inferior nasal region (RS17) is located nearer to the origin of retinal vessels, thereby protecting it from severe ischemic damage. These findings imply that DMI should be tracked using tailored criteria different from those for other retinal disorders.

Both MP3 and MAIA now have commercially available inbuilt standard 37 stimuli grids covering RS on various diameters ranging from 2° to 10° [18,36]. In the present study, we employed customized 21 stimuli within a 3 × 3 mm grid on MAIA (equivalent to a 9° diameter circle), and we analyzed data from DMI eyes without DME as our focus was to study the RS changes in the foveal and parafoveal areas. Therefore, our MP protocols differed from those used in DME [37].

The present study has several strengths worth considering. First, this study demonstrates the natural history of MP changes in DMI over one year using point-by-point measures for RS. Second, the definitions of DMI were based on OCTA metrics, which prevent bias generated from subjective evaluation. Finally, the study cohort was homogeneous, comprising only stable-treated PDR patients with DMI without DME, thereby avoiding confounding factors due to the range of DR severity.

We are also aware of the limitations of the study design and open to future research directions. For instance, we set a relatively good VA inclusion criterion to increase the chance of getting valid MP results. A study based on objective parameters, such as changes in OCTA for patients with poorer VA, may be considered. Moreover, MP was conducted only once in each visit; therefore, there was no test–retest variability to evaluate the validity of the results. Finally, longer follow-ups may be required to determine the extent of damage in DMI.

## 5. Conclusions

In conclusion, RS loss is a gradual process in DMI. While MP is a valuable tool for endpoint measurement in clinical trials targeting patients with DMI, the definitions of successful treatment should be tailored based on the natural history of DMI. Reducing RS losses of ≥2 dB in five random PWS may be a clinically meaningful outcome measure, especially in eyes with baseline oRS ≥ 25 dB.

## Figures and Tables

**Table 1:** Patient demographics and ocular characteristics at baseline.

**Demographics**	**All Participants (*n* =** **88** **)**
Age, mean (SD), years	58 (12)
Male, No. (%)	53 (60%)
T1DM, No. (%)	31 (35%)
T2DM, No. (%)	57 (65%)
Duration of diabetes, mean (SD), years	27 (14)
Ocular characteristics	All eligible eyes (*n* = 97)
BCVA, mean (SD), ETDRS letters	78 (8)
≥70 letters, No. (%)	85 (88%)
LLVA, mean (SD), ETDRS letters	67 (11)
≥70 letters, No. (%)	48 (50%)
LLD, mean (SD), ETDRS letters	11 (7)
Pseudophakia, No. (%)	42 (34%)
History of vitrectomy, No. (%)	15 (12%)
History of DME, No. (%)	22 (18%)

Abbreviations: BCVA = best-corrected visual acuity; DME = diabetic macular edema; ETDRS = Early Treatment of Diabetic Retinopathy Study; LLD = low-luminance deficiency; LLVA = low-luminance visual acuity; SD = standard deviation; T1DM = type 1 diabetes mellitus; T2DM = type 2 diabetes mellitus.

**Table 2:** Microperimetry changes in diabetic macular ischemia over time.

*n* = 97	Baseline Mean (SD), dB	At One Year Mean (SD), dB	Changes	*p* Value Clustered ^a^
Overall RS	23.7 (4.2)	23.9 (3.9)	0.3 (2.6)	0.33
<25 dB No. (%)	50 (52%)	50 (52%)	0 (0%)	-
Parafoveal RS	24.0 (4.3)	24.2 (4.0)	0.2 (2.9)	0.54
Regional sensitivity				
MS1 (central)	22.5 (5.0)	22.9 (4.9)	0.4 (2.9)	0.21
MS2 (nasal)	24.9 (4.5)	25.0 (4.6)	0.1 (3.8)	0.79
MS3 (superonasal)	24.5 (5.2)	25.2 (4.4)	0.7 (4.1)	0.12
MS4 (superior)	24.2 (5.9)	24.5 (4.8)	0.4 (3.4)	0.33
MS5 (superotemporal)	24.7 (3.9)	24.6 (4.1)	−0.1 (3.6)	0.86
MS6 (temporal)	23.8 (4.9)	23.9 (5.4)	0.1 (4.8)	0.82
MS7 (inferotemporal)	22.8 (5.4)	23.4 (5.2)	0.6 (3.8)	0.12
MS8 (inferior)	23.6 (4.9)	24.0 (4.6)	0.4 (4.0)	0.34
MS9 (inferonasal)	24.4 (4.9)	24.1 (4.3)	−0.3 (4.0)	0.47

Abbreviations: dB = decibel; MS = mean sensitivity; RS = retinal sensitivity; SD = standard deviation. ^a^ Paired *t*-test with clustered standard errors to account for subject clustering.

**Table 3:** Percentage of retinal sensitivity loss in diabetic macular ischemia at one year.

MP at One Year Compared to Baseline (*n* = 97)	Loss ≥7 dB No. (%)	Loss ≥2 dB No. (%)	Any Loss No. (%)	Loss ≥7 dB in ≥5 Loci No. (%)	Loss ≥2 dB in ≥5 Loci No. (%)	Any Loss in ≥5 Loci No. (%)
Overall RS (Macula)	1 (1%)	10 (10%)	42 (43%)	4 (4%)	71 (73%)	71 (73%)
Parafoveal RS (Parafovea)	2 (2%)	13 (13%)	46 (47%)	1 (1%)	44 (45%)	44 (45%)
Regional sensitivity						
MS1 (central fovea)	1 (1%)	16 (16%)	40 (41%)	0 (0%)	2 (2%)	3 (3%)
MS2 (nasal)	2 (2%)	29 (30%)	41 (42%)	-	-	-
MS3 (superonasal)	4 (4%)	25 (26%)	31 (32%)	-	-	-
MS4 (superior)	2 (2%)	25 (26%)	39 (40%)	-	-	-
MS5 (superotemporal)	3 (3%)	24 (25%)	42 (43%)	-	-	-
MS6 (temporal)	7 (7%)	24 (25%)	35 (36%)	-	-	-
MS7 (inferotemporal)	3 (3%)	18 (19%)	33 (34%)	-	-	-
MS8 (inferior)	3 (3%)	25 (26%)	37 (38%)	-	-	-
MS9 (inferonasal)	1 (1%)	32 (33%)	49 (51%)	-	-	-
Pointwise sensitivity						
RS1	8 (8%)	39 (40%)	39 (40%)	-	-	-
RS2	5 (5%)	37 (38%)	37 (38%)	-	-	-
RS3	3 (3%)	34 (35%)	36 (37%)	-	-	-
RS4	3 (3%)	33 (34%)	35 (36%)	-	-	-
RS5	2 (2%)	36 (37%)	38 (39%)	-	-	-
RS6	8 (8%)	31 (32%)	31 (32%)	-	-	-
RS7	5 (5%)	33 (34%)	34 (35%)	-	-	-
RS8	7 (7%)	34 (35%)	36 (37%)	-	-	-
RS9	6 (6%)	30 (31%)	30 (31%)	-	-	-
RS10	2 (2%)	40 (41%)	40 (41%)	-	-	-
RS11	4 (4%)	34 (35%)	35 (36%)	-	-	-
RS12	1 (1%)	30 (31%)	31 (32%)	-	-	-
RS13	5 (5%)	31 (32%)	32 (33%)	-	-	-
RS14	6 (6%)	34 (35%)	34 (35%)	-	-	-
RS15	5 (5%)	33 (34%)	33 (34%)	-	-	-
RS16	1 (1%)	30 (31%)	32 (33%)	-	-	-
RS17	7 (7%)	45 (46%)	46 (47%)	-	-	-
RS18	2 (2%)	29 (30%)	31 (32%)	-	-	-
RS19	4 (4%)	38 (39%)	39 (40%)	-	-	-
RS20	6 (6%)	26 (27%)	27 (28%)	-	-	-
RS21	2 (2%)	35 (36%)	36 (37%)	-	-	-

Abbreviations: dB = decibel; MP = microperimetry; MS = mean sensitivity; RS = retinal sensitivity.

**Table 4:** A comparison of retinal sensitivity loss at one year between eyes with good and impaired retinal sensitivity at baseline using 25 dB as the cut-off.

Microperimetry No. (%) *n* = 97	Loss ≥7 dB at One Year			Loss ≥2 dB at One Year			Any Loss at One Year		
	Baseline Overall RS <25 dB *n* = 50	Baseline Overall RS ≥25 dB *n* = 47	*p* Value	Baseline Overall RS <25 dB *n* = 50	Baseline Overall RS ≥25 dB *n* = 47	*p* Value ^a^	Baseline Overall RS <25 dB *n* = 50	Baseline Overall RS ≥25 dB *n* = 47	*p* Value ^a^
Overall RS	0 (0%)	1 (2%)	NA ^b^	4 (8%)	6 (13%)	0.54	16 (32%)	26 (55%)	0.01
Parafoveal RS	0 (0%)	2 (4%)	NA ^b^	5 (10%)	8 (17%)	0.46	19 (38%)	27 (57%)	0.04
Regional sensitivity									
MS1	0 (0%)	1 (2%)	NA ^b^	8 (16%)	8 (17%)	0.90	18 (36%)	22 (47%)	0.24
MS2	1 (2%)	1 (2%)	NA ^b^	15 (30%)	14 (30%)	0.91	18 (36%)	23 (49%)	0.16
MS3	1 (2%)	3 (6%)	NA ^b^	11 (22%)	14 (30%)	0.34	14 (28%)	17 (36%)	0.35
MS4	1 (2%)	1 (2%)	NA ^b^	11 (22%)	14 (30%)	0.32	14 (28%)	25 (53%)	0.01
MS5	1 (2%)	2 (4%)	NA ^b^	12 (24%)	12 (26%)	0.90	18 (36%)	24 (51%)	0.10
MS6	4 (8%)	3 (6%)	0.63	12 (24%)	12 (26%)	0.77	17 (34%)	18 (38%)	0.66
MS7	0 (0%)	3 (6%)	NA ^b^	9 (18%)	9 (19%)	0.88	15 (30%)	18 (38%)	0.19
MS8	1 (2%)	2 (4%)	NA ^b^	16 (32%)	9 (19%)	0.11	22 (44%)	15 (32%)	0.21
MS9	0 (0%)	1 (2%)	NA ^b^	17 (34%)	15 (32%)	0.81	24 (48%)	25 (53%)	0.72
Pointwise sensitivity									
RS1	3 (6%)	5 (11%)	0.43	22 (44%)	17 (36%)	0.43	22 (44%)	17 (36%)	0.43
RS2	3 (6%)	2 (4%)	0.67	18 (36%)	19 (40%)	0.63	18 (36%)	19 (40%)	0.63
RS3	3 (6%)	0 (0%)	NA ^b^	17 (34%)	17 (36%)	0.83	19 (38%)	17 (36%)	0.88
RS4	3 (6%)	0 (0%)	NA ^b^	13 (26%)	20 (43%)	0.10	14 (28%)	21 (45%)	0.09
RS5	2 (4%)	0 (0%)	NA ^b^	20 (40%)	16 (34%)	0.46	21 (42%)	17 (36%)	0.47
RS6	6 (12%)	2 (4%)	0.28	17 (34%)	14 (30%)	0.70	17 (34%)	14 (30%)	0.70
RS7	3 (6%)	2 (4%)	0.67	14 (28%)	19 (40%)	0.26	15 (30%)	19 (40%)	0.41
RS8	6 (12%)	1 (2%)	NA ^b^	14 (28%)	20 (43%)	0.16	16 (32%)	20 (43%)	0.29
RS9	1 (2%)	6 (11%)	NA ^b^	12 (24%)	18 (38%)	0.14	12 (24%)	18 (38%)	0.14
RS10	1 (2%)	1 (2%)	NA ^b^	18 (36%)	22 (47%)	0.23	18 (36%)	22 (47%)	0.23
RS11	1 (2%)	3 (6%)	NA ^b^	16 (32%)	18 (38%)	0.47	17 (34%)	18 (38%)	0.59
RS12	0 (0%)	1 (2%)	NA ^b^	10 (20%)	20 (43%)	0.02	10 (20%)	21 (45%)	0.01
RS13	1 (2%)	4 (9%)	NA ^b^	14 (28%)	17 (36%)	0.30	15 (30%)	17 (36%)	0.41
RS14	4 (8%)	2 (4%)	0.50	13 (26%)	21 (45%)	0.03	13 (26%)	21 (45%)	0.03
RS15	3 (6%)	2 (4%)	0.70	15 (30%)	18 (38%)	0.37	15 (30%)	18 (38%)	0.37
RS16	1 (2%)	0 (0%)	NA ^b^	13 (26%)	17 (36%)	0.26	15 (30%)	17 (36%)	0.51
RS17	5 (10%)	2 (4%)	0.30	24 (48%)	21 (45%)	0.75	24 (48%)	22 (47%)	0.91
RS18	1 (2%)	1 (2%)	NA ^b^	13 (26%)	16 (34%)	0.38	15 (30%)	16 (34%)	0.68
RS19	4 (8%)	0 (0%)	NA ^b^	18 (36%)	20 (43%)	0.54	18 (36%)	21 (45%)	0.40
RS20	3 (6%)	3 (6%)	0.98	12 (24%)	14 (30%)	0.48	12 (24%)	15 (32%)	0.34
RS21	0 (0%)	2 (4%)	NA ^b^	14 (28%)	21 (45%)	0.09	15 (30%)	21 (45%)	0.13

Abbreviations: dB = decibel; MS = mean sensitivity; RS = retinal sensitivity. ^a^ *p*-values generated using GEE model (binomial family, logit link) accounting for clustering by subject. ^b^ Insufficient sample size.

**Table 5:** The predictability of pointwise retinal sensitivity loss in at least 5 loci at one year using baseline overall retinal sensitivity of 25 dB as a threshold.

Microperimetry Region No. (%) *n* = 97	Loss ≥7 dB in ≥5 Loci at One Year			Loss ≥2 dB in ≥5 Loci at One Year			Any Loss in ≥5 Loci at One Year		
	Baseline Overall RS <25 dB *n* = 50	Baseline Overall RS ≥25 dB *n* = 47	*p* Value	Baseline Overall RS <25 dB *n* = 50	Baseline Overall RS ≥25 dB *n* = 47	*p* Value ^a^	Baseline Overall RS <25 dB *n* = 50	Baseline Overall RS ≥25 dB *n* = 47	*p* Value ^a^
Macula (21 loci)	2 (4%)	2 (4%)	Na ^b^	33 (66%)	38 (81%)	0.10	33 (66%)	38 (81%)	0.10
Parafovea (12 loci)	0 (0%)	1 (2%)	Na ^b^	18 (36%)	26 (55%)	0.05	18 (36%)	26 (55%)	0.05
Central fovea (5 loci)	0 (0%)	0 (0%)	Na ^b^	1 (2%)	1 (2%)	Na ^b^	2 (4%)	0 (0%)	Na ^b^

Abbreviations: dB = decibel; RS = retinal sensitivity. ^a^ *p*-values generated using GEE model (binomial family, logit link) accounting for clustering by subject. ^b^ Insufficient sample size.

## Data Availability

Dr Sivaprasad has full access to all the data in the study and takes responsibility for both the integrity of the data and the accuracy of the data analysis. The data will be made available upon request (sobha.sivaprasad@nhs.net).
